# High class filtering facepiece (FFP) are fundamental and effective in protection of emergency health care workers: an observational cohort study in a German community

**DOI:** 10.1186/s13049-021-00969-0

**Published:** 2021-10-30

**Authors:** Martin Lier, Stefan Nessler, Christine Stadelmann, Meike Pressler, Leif Saager, Onnen Moerer, Markus Roessler, Konrad Meissner, Martin S. Winkler

**Affiliations:** 1 Department of Anesthesiology, Emergency and Intensive Care Medicine, University Medical Centre Göttingen, Robert-Koch-Str. 40, 37075 Göttingen, Germany; 2 Institute for Neuropathology, University Medical Centre Göttingen, Robert-Koch-Str. 40, 37075 Göttingen, Germany

**Keywords:** Personal protection equipment, Filtering facepiece, FFP2, N95, SARS-CoV-2, Seroprevalence, Emergency medical services

## Abstract

**Background:**

Severe acute respiratory syndrome coronavirus-2 (SARS-CoV-2) is a highly contagious airborne virus inducing pandemic coronavirus disease 2019 (COVID-19). This is most relevant for medical staff working under harmful conditions in emergencies often dealing with patients and an undefined SARS-CoV-2 status. We aimed to measure the effect of high-class filtering facepieces (FFP) in emergency medical service (EMS) staff by analyzing seroprevalence and history of positive polymerase chain reaction (PCR) for SARS-CoV-2.

**Method:**

This observational cohort study included workers in EMS, who were compared with hospital staff (HS) and staff, which was not directly involved in patient care (NPC). All direct patient contacts of EMS workers were protected by FFP2/N95 (filtering face piece protection class 2/non-oil-based particulates filter efficiency 95%) masks, whereas HS was protected by FFP2/N95 exclusively when a patient had a proven or suspected SARS-CoV-2 infection. NPC was not protected by higher FFP. The seroprevalence of SARS-CoV-2 antibodies was analyzed by *immunoassay* by end of 12/2020 together with the history of a positive PCR. In addition, a self-assessment was performed regarding the quantity of SARS-CoV-2 positive contacts, about flu symptoms and personal belief of previous COVID-19 infections.

**Results:**

The period in which contact to SARS-CoV-2 positive patients has been possible was 10 months (March to December 2020)—with 54,681 patient contacts documented for EMS—either emergencies (n = 33,241) or transportation services (n = 21,440). Seven hundred-thirty (n = 730) participants were included into the study (n = EMS: 325, HS: 322 and NPC: 83). The analysis of the survey showed that the exposure to patients with an unknown and consecutive positive SARS-CoV-2 result was significantly higher for EMS when compared to HS (EMS 55% vs. HS 30%, *p* = 0.01). The incidence of a SARS-CoV-2 infection in our cohort was 1.2% (EMS), 2.2% (HS) and 2.4% (NPC) within the three groups (ns) and lowest in EMS. Furthermore, the belief of previous COVID-19 was significant higher in EMS (19% vs. 10%),

**Conclusion:**

The consistent use of FFP2/N95 in EMS is able to prevent work-related SARS-CoV-2 infections in emergency situations. The significance of physical airway protection in exposed medical staff is still relevant especially under the aspect of new viral variants and unclear effectiveness of new vaccines.

**Graphical Abstract:**

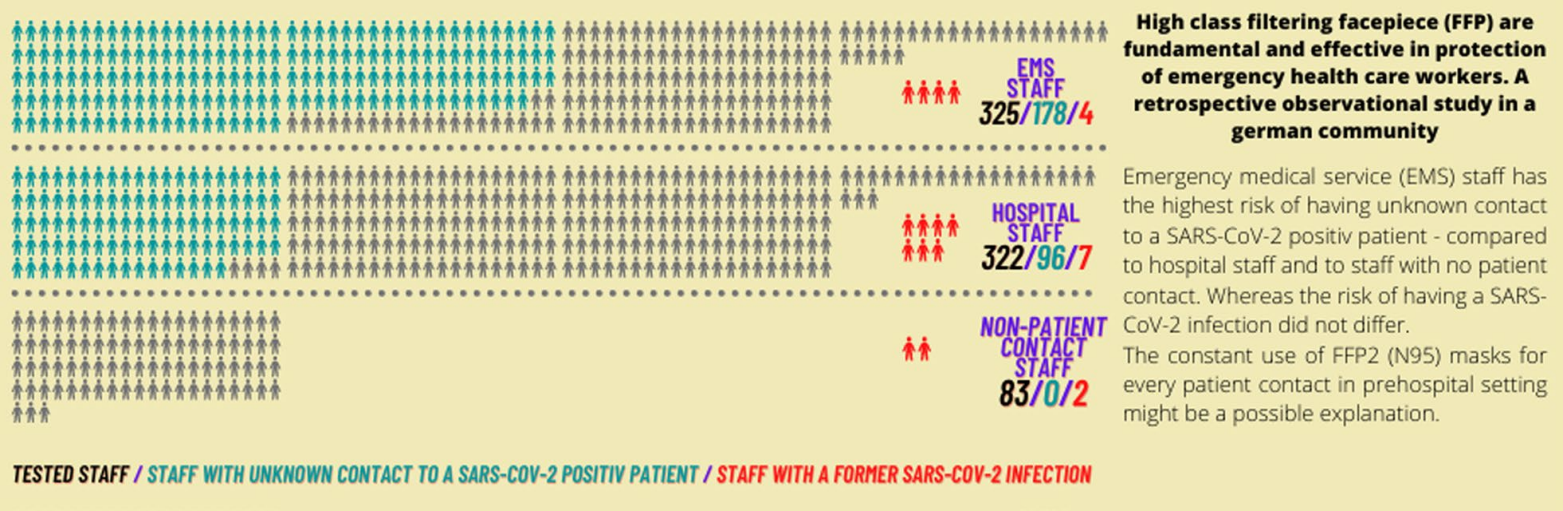

## Introduction

Patient care in emergencies is challenging even for specialized and trained emergency medical service (EMS) often treating critically ill patients under adverse working conditions. During the current severe acute respiratory syndrome coronavirus-2 (SARS-CoV-2) pandemic adverse working condition were even worse due to the fact that EMS staff are constantly confronted with a viral hazard while treating potentially infected patients. Indeed, the increased risk of work-related infections within health-care workers has been previously addressed by other researches showing a SARS-CoV-2 antibody (Ab) seroprevalence up-to 4.04%, which is clearly higher compared to the expected incidence in controls [1, 2]. In these studies, antibody titers of health-care workers were adjusted to the medical discipline and always increased in EMS staff compared to other disciplines not regularly involved in emergency medicine [1, 2]. One cannot ignore that an increased exposure to highly contagious SARS-CoV-2 is a consistent psychological stress factor in particular for EMS staff treating patients with an unclear disease status. Even though the number of vaccinated EMS workers increases reports of vaccination failure arise [3–5]. In future, the appearance of new aggressive and escape variants such as B.1.1.7, P.1 and B1.617 *plus* an unclear duration of protective effect of vaccines sustain a high level of insecurity for involved EMS personnel.

In this context, personal protective equipment (PPE) remains the most important tool to minimize viral exposure notably under working conditions characterized by urgency, contact to highly infectious airway material and in overcrowded environments of accident and emergency (A&E) departments or ambulance vehicles. By definition risk adjustment in those situations can be made of the potential *patient-to-staff* contact with: (a) patients with known SARS-CoV-2 infection *or* suspected infection due to positive symptoms and (b) patients with unknown status. The primary aim of this study was to evaluate the effectiveness of PPE while treating the described patient groups under emergency situations compared to controlled situations in intensive care units (ICU) and operation theatre (OT).

## Methods

### Study design and setting

We measured the effectiveness of filtering facepieces (FFP) as a basic element of PPE in outpatient emergency settings to avoid SARS-CoV-2 infection. We use a retrospective-observational cohort study design to determine the incidence of SARS-CoV-2 infections, by determination of seral SARS-CoV-2 antibodies (SARS-CoV-2 ab) in emergency medical service staff (EMS). This methodology has been previously described by other research groups and therefore seemed adequate for our aim [6, 7]. To answer the question, if the specific risk of EMS was higher when compared to various other health care professionals, we further measured seral SARS-CoV-2 ab in two comparison groups: I) Hospital staff (HS) working in intensive care (ICU) or operation theatre (OT) and II) staff, who was not involved in patient care but working in general hospital services, such as transportation (NPC). A “positive” infection was defined by detection of seral SARS-CoV-2 ab (sero-positive) or when the study participants presented a positive SARS-CoV-2 polymerase chain reaction (PCR) test within the study period from (March 2020 to December 2020). In addition, specific risk exposure to SARS-CoV-2 was addressed by a self-designed structured interview. All participants had to answer five questions (Q) by *yes/no* and should report the number of contacts, when applicable: (Q1) “*Were you aware of any conscious and known contact to a SARS-CoV-2 positive patient or a patient with a suspected infection?*” and (Q2) “*Were you aware of any unconscious and unknown contact to a SARS-CoV-2 positive patient with an unclear SARS-CoV-2 status at time of treatment or contact?*” If Q2 was answered positive, we asked Q3: “*In terms of unknown contact did you used a surgical mask or was your contact unprotected?*” We further evaluate the individual COVID-19 status of all study participants by asking: (Q4) “*Did you ever had flu like symptoms since the beginning of this on-going pandemic?*” and (Q5) *“Do you think you are/ were infected with the SARS-CoV-2 (COVID-19)?”* The questions were asked at the same visit with venipuncture to avoid an overlap of possible infection with the question based self-assessment. All participants included were ≥ 18 years. Written informed consent has been obtained from all individuals. The study protocol was approved by the local ethic committee of the University Medical Center Göttingen (Ref. #8/9/20).

**Fig. 1:**
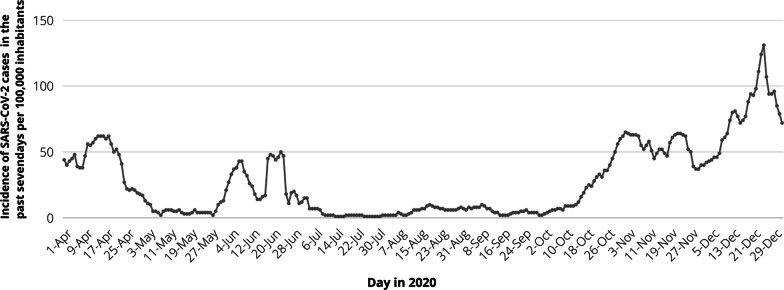
Incidence of SARS-CoV-2 positive cases in the metropolitan area of Göttingen. The incidence within the past 7 days presented as cases/100,000 inhabitants. Three pandemic waves were reported with peaks in April, June and December 2020. By December 2020 1.02% of the population has been infected with SARS-CoV-2 [Personal communication with the local public health department Göttingen]

The administrative district of Göttingen in which the study was conducted has approx. 326,000 citizens with 119,000 citizens living in the city area of Göttingen. The regional EMS employs approx. 400 people in prehospital care. From the first identification of SARS-CoV-2 until end of the study (beginning of Mar. until Dec. 2020, 10 months) 33,241emergencies and 21,440 transport have been performed. During observation 3342 positive infections have been reported in the metropolitan area (incidence rate for SARS-CoV-2 infection of 1.02%, Fig. 1) [8].

### Personal protective strategy in emergency medical service staff, Hospital staff and non-patient contact staff

One hypothesis of this study was that emergency medical service staff (EMS) has a higher exposure to SARS-CoV-2 and that the number of SARS-CoV-2 positive individuals is increased when compared to other groups. The defined groups EMS, hospital staff (HS) and non-patient contact staff (NPC) worked under different protective strategies (Table 1). Mandatory for EMS staff was an airway protection with FFP2/N95 (filtering face piece protection class 2/Non-oil based particulates filter efficiency 95%) masks by official order from the beginning of this observation in March 2020. FFP2/N95 masks have filter performance of > 94%. Surgical overall, hood and protective glasses were mandatory when in contact with known positive cases or when a SARS-CoV-2 infection was highly suspected defined by national guidelines published by the national institute for infectious disease control [9]. A FFP3/N99 (filtering face piece protection class 3/Non-oil based particulates efficiency 99%) mask with filter performance > 98% was required upon invasive, airway management procedures such as intubation. Surgical masks hat to be worn on demand between patient contacts. For HS PPE guidelines were identical as for EMS workers except for the requirement of FFP2/N95 masks in general and in all patient-to-staff contacts and NPC was protected by surgical masks. Table 1 summarizes the different groups compared and the PPE strategies.

**Table 1:**
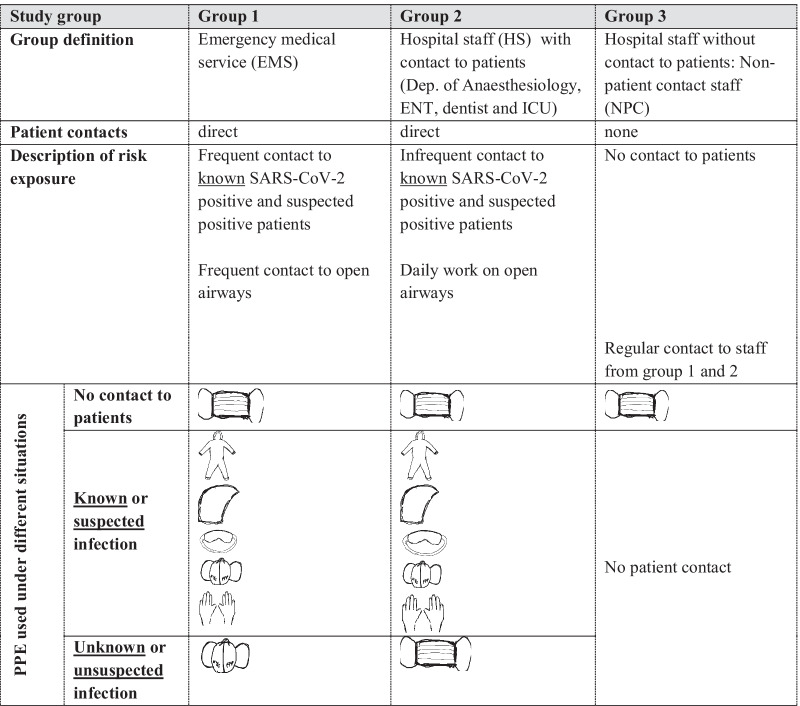
Summary of group definition by risk exposure and the used personal protection equipment

surgical mask;  FFP2/N95 or FFP3/N99 face mask;  protective gown;

protective head;  glasses;  gloves; *EMS*: emergency medical service, *HS* hospital staff, *NPC* non-patient contact staff, *ENT* ear neck throat specialist, *ICU* intensive care unit

### Identification of SARS-CoV-2 infection and statistical analysis

IgG serum antibody reactivity against SARS-CoV2 spike S1 at 1:50 serum dilution was evaluated by a cell based assay using HEK293 (Human Embryonic Kidney) cells stably transfected with either the pCMV3-2019-nCoV-S1 (HEK^spike S1^, Sino Biologicals VG40591-UT, China) or the pCMV-untagged (HEK^EV^, Sino Biologicals CV011) expression plasmids as described previously [10]. Serum samples were rated antibody positive if the median fluorescence intensity ratio (MFI) of the sample exceeded the MFI ratio of prepandemic controls + 5 SD.

Statistical analysis was conducted using SPSS (version 26; IBM) and group differences we calculated by non-parametric Kruskal–Wallis test.

## Results

Sevenhundred-thirty (n = 730) participants were included in this study. Three groups were defined (Tables 1, 2): EMS staff (n = 325) *with* direct involvement in emergency patient care, HS (n = 322) from the ICU (nursing staff 126; physicians 39) and OT (nursing or assistant staff 110; physicians 47) *with* direct involvement in patient care including staff from anaesthesiology department, ear-nose-throat (ENT) specialists, dentist/oral surgeons and associated nursing staff. NPC (n = 83) was *not* involved in patient care by definition. Each group faced a different risk profile regarding SARS-CoV-2 exposition and is described in Table 1. EMS staff was frequently, directly exposed to positive patients and was either aware or unaware of patients individual SARS-CoV-2 status. Working conditions for EMS staff are best described by “close contact” and often includes procedures involving the upper airways. In contrast, HS was more often aware of SARS-CoV-2 positive patients. In summary, the SARS-CoV-2 status of hospital patients was more often defined and safety was higher for HS. According to the expected risk the PPE was adjusted and is described in Table 1: While off duty a surgical masks were worn in all groups, the main difference was the constant protection by higher class FFP masks at any time in EMS (Table 1).

**Table 2:** Results from survey/self-assessment and serological and PCR screening

Parameter	All	EMS	HS	NPC	*p *value
Study participants (n)	730	325	322	83	N/A
*Risk exposure*					
Q1: Known contact to SARS-CoV-2 positive patient *or* suspected SARS-CoV-2 patient, n (%)	473 (65)	276 (85)	197 (61)	N/A	0.01^#^
Q2: Unknown contact to SARS-CoV-2 positive patient, n (%)	275 (38)	178 (55)	96 (30)	N/A	0.01^#^
Q3: In terms of unknown contact: PPE used was a surgical mask or unprotected, n (%)	74 (27)	15 (8)	58 (60)	N/A	< 0.01^#^
*Awareness of symptoms and COVID-19*					
Q4: Flu symptoms within last 10 months, n (%)	360 (50)	154 (47)	169 (52)	37 (44)	0.31^##^
Q5: Self assessment: Do you think you have been infected with SARS-CoV-2 or COVID-19, n (%)	83 (16)	61 (19)	34 (10)	8 (10)	0.02^##^
*Seroprevalence SARS-CoV-2 ab or PCR*^§^					
Sero + *and* PCR + , n	6	2	3	1	ND
Sero + *but* PCR − , n	3	1	1	1	ND
PCR + *but* Sero − , n	4	1	3	0	ND
n positive/ n paticipants	13/730	4/325	7/322	2/83	ND
*SARS-CoV-2 incidence*					
Incidence, %	1.8	1.2	2.2	2.4	0.58^##^

*Q* question, *EMS* emergency medical service, *HS* hospital staff, *NPC* non-patient contact group, *PPE* personal protective equipment, + positive and – negative, *N/A* not applicable, *ND* not determined

^§^Positive PCR test result provided by study participants and within the observational period (March until December 2020)

^#^Student *t* test comparing EMS with HS

^##^ANOVA Kruskal–Wallis test for trend in all groups

Results from the survey and self-assessment are presented in Table 2. Five questions (Q) were asked. Q1: 85% of EMS and 61% of HS and none of the study participants in NPC quoted contacts to SARS-CoV-2 positive or suspected positive patients. This underlies a consistent classification of risk profiles within the observed groups. (Q2) We determined a similar result regarding unknown contacts: The number of unknown contacts to SARS-CoV-2 positive patients in EMS was significant higher when compared to HS (Table 2, *p* < 0.01). In Q3 all study participants where asked for the PPE used during the described situation in Q2. While 8% of EMS was protected by surgical masks or less this was significantly higher in HS (Table 2). Taken together, exposition of EMS to SARS-CoV-2 was higher but happened under better protection.

We further evaluted the awareness of symptoms in Q4 and Q5 (Table 2). In all groups more then 40% of reported flu symptoms within the observational period. Interestingly, the question about previous COVID-19 was answered positive by 19% in EMS and significantly higher when compared to HS and NPC (Table 2, *p* = 0.02).

**Fig. 2:**
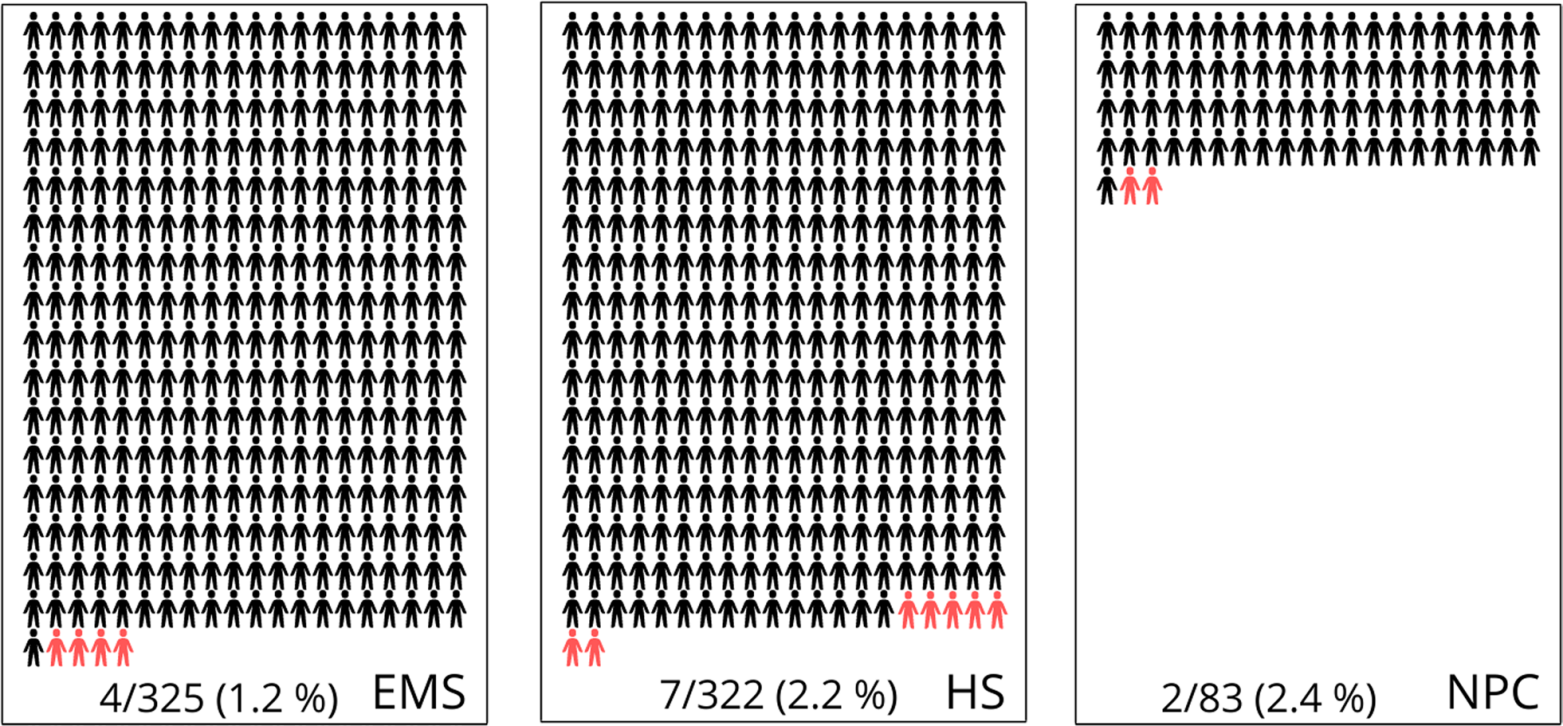
Incidence of SARS-CoV-2 within groups. Positive cases are marked in red and were defined either by positive viral polymerase chain reaction (PCR) within the observational period (March–December 2020) and/or detection of seral antibodies (Ab) against SARS-CoV-2. *EMS* emergency medical service staff, *HS* hospital staff, *NPC* non-patient contact staff

Aim of this study was the determination of work-related SARS-CoV-2 infection (seroprevalence or postive PCR) in EMS compared to HS and NPC compared to the overall SARS-CoV-2 incidence within the EMS covered region. Figure 1 shows the incidence in the adminstrative district of Göttingen over the past 10 months. The Robert-Koch-Institute (RKI) uses the keyfigure cases/100.000 citizens over a period of 7 day to report the nationwide viral spread: In March 2020 this 7-day incidence reached a maximum of 40 cases per 100,000 and 130 cases per 100,000 in December. In addition, EMS performed approx. 54,600 emergencies or transportation services within the observational period and therorectically this accounts for 270 patient contacts/ EMS member (2 EMS members per deployment: 2 × 54,600/400) ignoring that we have only 81% of all EMS employees included. Our survey was able to detect four individuals with PCR positive results but negative SARS-CoV-2 ab and three sero-positive individuals, who were not aware of SARS-CoV-2 infection (PCR negative). Taken together, in December 2020 after 10 months of SARS-CoV-2 exposition and without an available vaccination: The work-related infection rate was 1.2% in EMS and lower when compared to HS (2.2%) and NPC (2.4%, Table 2 and Fig. 2 not significant).

## Discussion

Working in emergency situations during this on-going pandemic is a risk factor for airborne SARS-CoV-2 infection. We evaluated the strategy of PPE in EMS compared to a mixed cohort of health-care workers from ICU, OT staff and staff not involved in patient care. We found no difference for previous infection with SARS-CoV-2 within the reported groups. Interestingly, EMS has a significant higher risk of getting in contact with SARS-CoV-2 infected patients but seroprevalence was lower compared with HS. Our hypothesis that EMS had a higher SARS-CoV-2 exposure risk was true. However, this was not accompanied with an increased SARS-CoV-2 incidence and for this reason our hypothesis needs to be rejected.

The use of FFP2/N95 masks (with a filter performance of > 94%) or higher seems a persuasive and simple strategy to prevent airborne viral infection, which is still under debate compared to several other promoted strategies in the emergency medical services. For example, in Milan a specialized EMS team screened patients for the likelihood of patients being infected with SARS-CoV-2 before sending the medical response team [11] and during the SARS pandemic 2003 reports of reorganized EMS according to patients’ risk levels have been published [12]. Those strategies in emergency care presuppose an immediate diagnostic screening, which is problematic while facing COVID-19—a new disease entity with many asymptomatic patients and a long period of being infectious without having symptoms (1–3 days before symptoms) [13]. Furthermore, team allocations into response teams treating either infectious or non-infectious patients requires substantially more personnel. Under these aspects our study shows that a strategy with mandatory protection using FFP2/FFP3 is effective and efficient.

Efficient allocation of medical staff has already been problematic before this pandemic but this on-going disaster revealed a variety of new pandemic specific challenges: (1) increased rates of sick employees, (2) increase in quarantine times and (3) substantially higher working load for the employees on duty. Indeed, epidemics are associated with significant increased sick leave rates of employees. During the SARS outbreak in Toronto 2003 more than 430 EMS employees were at least one-time in quarantine (≥ 50%) and in New York last year 41% were not allowed to work due to contacts of suspected or confirmed SARS-CoV-2 infections [14, 15]. This has dramatic consequences on the work load for the remaining staff and is well reflected by the reported maximum sick leave rate of 20% in New York. Together, with an average quarantine duration between 20 and 25 days the cumulative “sick-leave” emerged a never seen degree [15].

For another reason the responsibility of the employers and authorities to establish sufficient, effective and safe preventive strategies are important: Health care workers in EMS and ICU fear do to get infected. Although we cannot provide a causal relationship between this undefined fear and the increased sick leave rates, a positive correlation can be assumed. Indeed, other studies have addressed this problem showing increased number of sick-leave rates during pandemics, which are independent from the time spend in quarantine due to positive contacts [16]. This is indirectly supported by our data showing an increased awareness for COVID-19symptoms: ≥ 45% of all participants positively replied to the question about flu symptoms during the last 10 months (Table 2). Together with up-to 20% being convinced of being infected with SARS-CoV-2 or having had COVID-19 respectively our data may strengthen the impression that this fear is real for the involved employees.

In conclusion, health care workers during pandemics are challenged by several stressors, which might be classified in (1) extrinsic such as quarantine time of co-workers with the consequence of increased work load for the remaining staff, and (2) intrinsic factors such as the higher sensitivity for COVID-19 like symptoms and fear of getting infected. The vicious circle out of *quarantine—reduced staff—increased work load—physical exhaustion—higher awareness of symptoms—continuous psychological stress* might effectively be interrupted by three easy procedures: Risk reduction by mandatory high-class PPE, hygiene training and reduction of psychological stress by continuous testing.

A limitation of this study is the comparably low incidence rate in our area with 3346 positive cases in 330,000 inhabitants (1.01%) from March until December 2020 [17]. However, the epidemic in 2003 in Taiwan with an even lower incidence of 0.01% revealed 100-times higher infections in EMS [12]. Even though no data exist about PPE in this study these data underline an effective prevention of airborne corona viruses in our presented EMS group compared to the situation in 2003. In addition, we did not only determine SARS-CoV-2 ab in serum but expanded our observation by self-assessment of positive PCRs within the observational period. Considering an undefined period of detectable SARS-CoV-2 ab in serum we assume that our methods are able to present a realistic view of work-related infections [18–20]. Importantly, we can only overview work related factors contributing to the seroprevalence and cannot assess individual behavior during recreational and off-duty time. However, with a study population of 730 study participants we believe that those factors are equally distributed in all groups. A follow-up study should address this point in an extended survey.

## Conclusion

In conclusion, the reported results are promising in a pandemic phase with more and more individuals receiving vaccination on the one hand but under the aspect of new aggressive virus variants on the other hand. We demonstrate that a risk adjusted PPE is safe and efficient to limit risk exposure to SARS-CoV-2.

## Data Availability

The datasets of the study are available from the corresponding author on request.
